# An Investigation of Virtual Reality Nature Experiences in Patients With Metastatic Breast Cancer: Secondary Analysis of a Randomized Controlled Trial

**DOI:** 10.2196/38300

**Published:** 2022-07-22

**Authors:** Stanley Chin, Alana Cavadino, Amelia Akroyd, Geraldine Tennant, Rosie Dobson, Adele Gautier, Lisa Reynolds

**Affiliations:** 1 Department of Psychological Medicine The University of Auckland Auckland New Zealand; 2 Section of Epidemiology and Biostatistics The University of Auckland Auckland New Zealand; 3 National Institute for Health Innovation The University of Auckland Auckland New Zealand; 4 Breast Cancer Foundation NZ Auckland New Zealand

**Keywords:** metastatic breast cancer, virtual reality, nature connectedness, intervention, quality of life

## Abstract

**Background:**

Connection with nature has well-established physical and psychological benefits. However, women with metastatic breast cancer (MBC) are often unable to access nature because of physical limitations, psychological barriers, and treatment demands. Virtual reality (VR) nature experiences offer an alternative means of connecting with nature and may be of particular benefit to patients with cancer who are house- or hospital-bound.

**Objective:**

This study aims to explore whether VR nature experiences are associated with physical and psychological benefits for women with MBC who are disconnected with nature.

**Methods:**

This secondary analysis of a previous randomized controlled crossover trial recruited participants from the emailing lists of breast cancer support organizations. Participants were provided VR headsets for daily use in their homes for over 3 weeks. In the first week, participants used 1 of 2 VR nature experiences (*Ripple* or *Happy Place*) daily, followed by a 1-week washout period, before using the other VR experience every day for the final week. Outcomes assessed changes between baseline and postintervention scores in quality of life (EQ-5D-5L), pain (Brief Pain Inventory Short Form), fatigue (Functional Assessment of Chronic Illness Therapy-fatigue), depression (Depression, Anxiety, and Stress Scale-depression), anxiety (Depression, Anxiety, and Stress Scale-anxiety), and spiritual well-being (Functional Assessment of Chronic Illness Therapy- Spiritual Well-being) and investigated whether benefits were greater in participants who were not strongly connected with nature at baseline.

**Results:**

A total of 38 women with MBC completed the VR interventions and were included in the analyses. Participants reported significantly less fatigue (*P*=.001), less depression (*P*<.001), and greater quality of life (*P=*.02) following the interventions than at baseline. Women with a weaker connection to nature reported greater fatigue (*P*=.03), depression (*P*=.006), and anxiety (*P*=.001), and poorer spirituality (*P*=.004) than their strongly connected counterparts. Only those with a weaker baseline connection with nature showed improvements in depression following the intervention (*P*=.03), with similar trends observed in fatigue (*P*=.07) and quality of life (*P*=.10).

**Conclusions:**

This study provides preliminary evidence that feeling connected with nature is associated with better physical and psychological status in patients with MBC and that VR nature interventions might be beneficial for this clinical population. Future studies should focus on activities that encourage connection with nature (rather than simply exposure to nature) and investigate the aspects of VR nature interventions that have the greatest therapeutic potential.

**Trial Registration:**

Australian New Zealand Clinical Trials Registry ACTRN12619001480178; https://tinyurl.com/et6z3vac

## Introduction

### Background

Metastatic breast cancer (MBC) is a terminal diagnosis that occurs when cancer cells spread from the breast to the lymph nodes and more distant regions such as the bone, brain, liver, and lung [1]. The impact of metastatic disease and its associated treatments (chemotherapy, radiotherapy, surgery, etc) can be physically and psychologically demanding. Although less research has been conducted on the effects of MBC compared with early disease [2], a limited body of work has identified physical challenges including pain and fatigue [3] and psychological problems including anxiety [4], depression [5], and spiritual distress [6]. Problematic physical and psychological issues are associated with diminished quality of life [7]. Recent data indicate that the median survival time following a diagnosis of MBC across all ages is 25 months [8]. Maintaining quality of life is particularly important in the context of a shortened life span. Therefore, pragmatic interventions that support the physical and psychological well-being of women with MBC are needed. This work investigates whether virtual reality (VR) nature experiences might offer benefit in this context.

Connecting to nature has well-established therapeutic benefits. For instance, exercising in the countryside improves mood [9], gardening promotes stress recovery [10], and immersing oneself in nature through activities such as “forest bathing” offers benefits to both mind and body [11] and can promote feelings of awe, wonder, and spiritual well-being [11,12]. Notably, studies have shown that exposure to specific elements of nature (eg, auditory and visual cues) can also be beneficial. Sounds of nature such as flowing water and birdsong are associated with improved stress recovery in healthy volunteers [13] and in clinical populations undergoing medical procedures [14]. Likewise, viewing images of natural landscapes during exercise is associated with improved mood and reduced blood pressure [15]. Specifically, qualitative work has proposed that connection with nature provides an enriching experience in which patients with cancer can source strength and meaning [16]. The concept of connection with nature has been assessed in a variety of ways, including the 21-item “nature relatedness scale” [17], the 14-item “connectedness to nature scale” [18], and the single item “inclusion of nature with self scale” [19]. The latter is not only brief but also appears particularly associated with well-being [20,21].

Exposure to nature through virtual means may offer proximal benefit where real-world exposure is not feasible. A VR experience is one in which an individual is immersed in, and interacts with, a computer-generated environment using a headset that displays visual and auditory stimuli that simultaneously obstructs the views and sounds of what is happening in the real-world context [22,23]. VR experiences can be wide-ranging, and nature-based experiences seem to have therapeutic potential. Experimental work has demonstrated that virtual exposure to nature offers more benefits than virtual urban environments [24]. A recent review noted the therapeutic benefits of VR nature experiences in psychiatric and medical care [25]. Notably, a study found that a VR nature video offered equivalent benefits to immersion in an actual real-world nature setting [26]. Thus, virtual experiences of nature offer promise in contexts in which people may be unable to connect directly with real-world nature.

Women with MBC face barriers to getting outdoors. The physical limitations of advanced cancer can reduce mobility [27-29], psychological issues such as low mood or demoralization can reduce the motivation to venture outside [30,31], and treatment demands can keep people tied to urban environments [32,33]. Thus, connecting with nature can be difficult for several reasons. Of relevance, research in the general population demonstrates a dose response such that the less time a person spends outdoors and the less vegetation in their neighborhood, the greater the psychological difficulties, even when controlling for sociodemographic factors [34]. Thus, connecting with nature through virtual means may be beneficial for women with MBC who are not currently connected with nature.

Although VR interventions have been studied during cancer treatment as a form of distraction [35,36] and nature-inspired VR experiences have been used specifically with patients undergoing chemotherapy [37], VR nature experience has never been investigated as an intervention in a patient’s own home. Home-based interventions play a role in addressing disparities in the uptake, adherence, and accessibility of psychological interventions for women with MBC [38]. Interventions that have the flexibility to be self-directed and delivered in a person’s home seem well suited to address such disparities.

### This Study

This study presents a secondary analysis of a randomized controlled trial comparing 2 VR nature interventions in women with MBC. Primary analyses, including a detailed discussion of the differences between the 2 interventions, are presented elsewhere [39]. The focus of this work was to assess whether VR nature experiences might be of greater benefit to women with MBC who are disconnected with nature than those who are connected with nature. We hypothesized that daily use of VR nature interventions in women with MBC would improve quality of life, reduce physical symptoms (pain and fatigue), and improve psychological well-being (depression, anxiety, and spiritual well-being) and that benefits would be moderated by baseline connection with nature. That is, we hypothesized that women who did not initially feel connected with nature would have worse quality of life, physical symptoms, and psychological well-being at baseline and would benefit more from VR exposure to nature than women who were initially highly connected with nature.

## Methods

### Research Design

This study reports secondary analyses from a randomized controlled crossover design in which participants were randomized to a different order of exposure to 2 VR nature experiences. A detailed discussion of the methods used in the original study has been published previously [39].

### Ethics Approval

This study was approved by the Health and Disability Ethics Committees (19/NTB/146) and registered on the Australian New Zealand Clinical Trials Registry (ACTRN12619001480178). Written informed consent was obtained from the participants, and all procedures were in accordance with the ethical standards of the responsible committee on human experimentation (institutional and national) and the Declaration of Helsinki 1975, as revised in 2000.

### Participants

Participants were recruited between October 2019 and March 2020 by advertising flyers sent to the emailing lists of 2 breast cancer support organizations. A total of 46 participants contacted the researchers and were assessed for eligibility. Women who could read and write English were included if they had a self-reported MBC diagnosis and (1) had experienced pain, fatigue or anxiety in the week before recruitment and (2) were able and willing to use a VR headset for at least 10 minutes a day for the study duration. Exclusion criteria were the presence of any visual, hearing, or cognitive impairments or face, head, or neck discomfort that would preclude them from wearing the VR headset.

### VR Equipment

**Figure 1:**
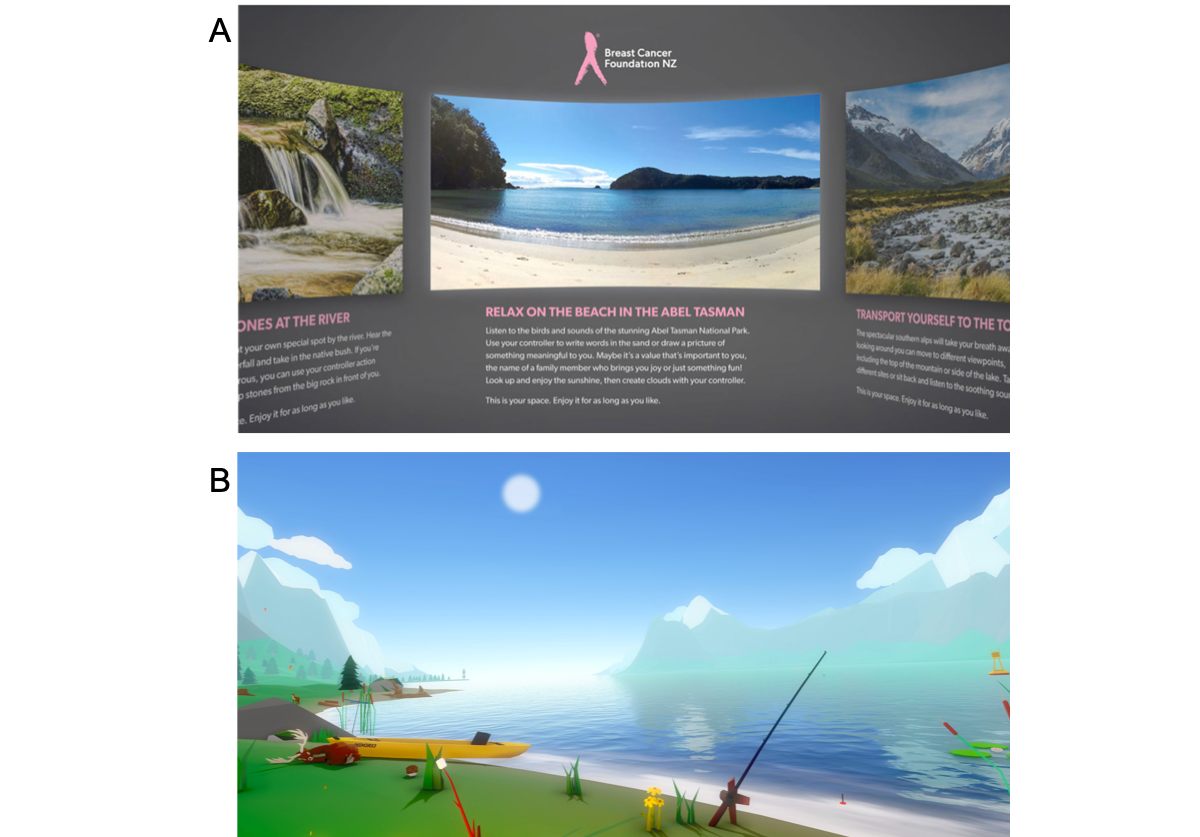
Images from Ripple (A) [40] and Happy Place (B) [41].

Participants were sent VR equipment and instructions via courier after obtaining study information and informed consent. The equipment included a Pico Goblin VR headset, remote control, headphones, charger and cable, batteries, screen-cleaning cloth, and a logbook to record daily use. The participants also received 4 envelopes with instructions to open them weekly, in a specified order. Each headset had 2 VR experiences installed: a real-world nature experience—*Ripple* and an animated experience—*Happy Place* (Figure 1 [40,41]).

*Ripple* is a real-world nature VR experience developed by Mixt Studio [40] and commissioned for the study by the Breast Cancer Foundation New Zealand based on feedback from qualitative work with patients with MBC. In this virtual experience, the sounds and images of three 360° nature environments are presented: (1) a view of different perspectives of a mountaintop, (2) a stacking stones activity by a waterfall, and (3) writing in the sand at a beach. The other VR experience was *Happy Place,* an animated nature VR experience developed by Hjärtat [41]. This experience involves a camping scene where participants can explore a campsite, listen to a guided relaxation exercise, and complete various activities such as roasting a marshmallow over a campfire or blowing bubbles.

### Procedure

After providing informed consent, participants completed baseline measures before being block randomized via REDCap (Research Electronic Data Capture; Vanderbilt University) by age (<50 years vs ≥50 years) to the order of exposure to each of the VR experiences. In one condition, participants used Ripple for 7 days, had a 7-day washout period, and then used the Happy Place for 7 days. The timings were the same in the other condition, but the order of exposure to the VR experiences was reversed. Participants were blinded to randomization and were instructed to use the headset for a minimum of 10 minutes per day during the periods of VR use. Primary analyses revealed no order effects or differences among the VR experiences [39]; therefore, these are not further reported in this work.

### Measures

Demographic and clinical information was collected at baseline, including age, ethnicity, education, relationship status, years since diagnosis, and current cancer treatment.

*Baseline connectedness to nature* was assessed using the Inclusion of Nature in the Self (INS) [19] measure. This single item presents a series of 7 diagrams with 2 circles that increasingly overlap; one circle represents the self and the other represents nature. Participants are asked to “mark the picture that best describes how close you have felt to nature in the past week,” with ratings ranging from 1 (circles do not overlap) to 7 (circles overlap entirely). Higher numbers represent a stronger self-perceived connection with nature. Given the aim of this work to assess whether VR exposure might differentially benefit women who were not initially connected with nature, we dichotomized scores at the point where there was potential to improve a person’s connection with nature, that is, where there was ≤50% overlap between the circles. Thus, scores between 1 and 5 were categorized as “weaker connection with nature” and 6 and 7 as “stronger connection with nature.” Given the lack of precedence in categorizing the INS into weak and strong connections with nature, we ran alternative models splitting the INS at other points as sensitivity analyses to evaluate this choice of cutoff on the results. As such, we also dichotomized the scores as weak (1-4) and strong (5-7), and then trichotomized the scores as weak (1-3), medium (4-5), and strong (6-7).

*Quality of life* was assessed using the EQ-5D-5L index score [42], which measures 5 dimensions of well-being: mobility, self-care, usual activities, pain or discomfort, and anxiety or depression. Participants chose from a 5-point Likert scale (1=“no problems” to 5=“unable to/extreme problems”). An external calculator based on the United Kingdom value set provided a value for the quality of life [43]. The EQ-5D-5L has shown good construct validity and reliability in patients with cancer [44]. Internal reliability was adequate for this study, both at baseline (Cronbach *α*=.82) and after the intervention (Cronbach *α*=.68).

*Fatigue* was measured using the Functional Assessment of Chronic Illness Therapy-fatigue (FACIT-fatigue [45]) scale. The scale assesses overall fatigue and its influence on daily activities and functioning in the past week and includes 13 items such as tiredness, weakness, and lack of energy. Participants rated the items on a 4-point Likert scale from 0 (“not at all”) to 4 (“very much”). The total fatigue score ranged from 0 to 52, with higher scores representing higher fatigue levels. In this study, FACIT-fatigue showed good internal reliability at baseline (Cronbach *α*=.91) and after the intervention (Cronbach *α*=.91).

The Brief Pain Inventory Short Form (BPI-SF [46]) measured *pain* over the past week. The first item asks participants to choose “yes” or “no” to whether they experienced pain other than everyday pain such as minor headaches. A total of 5 items measured pain levels in the past week, ranging from 0 (“no pain”) to 100 (“pain as bad as you can imagine”). The final 7 items assessed the level of interference of pain on well-being domains such as mood and sleep from 0 (“does not interfere”) to 100 (“completely interferes”). Excellent internal consistency has been demonstrated among patients with cancer [47]. This was similarly observed in this study: baseline Cronbach *α*=.93 and postintervention Cronbach *α*=.93.

*Depression* and *anxiety* were measured using the Depression, Anxiety, and Stress Scale Short Form (DASS-21) [48]. The DASS-21 is a 21-item measure that assesses anxiety, depression, and stress symptoms. Severity scores were calculated for each subscale, with higher scores indicating a greater severity. Each subscale has cutoff scores from “normal” to “extremely severe.” The DASS-21 has shown good internal consistency among patients with cancer (Cronbach *α*=.74−.91 [49]). In this study, the Depression, Anxiety, and Stress Scale (DASS)-depression subscale demonstrated excellent internal reliability before (Cronbach *α*=.94) and after intervention (Cronbach *α*=.89). Initial analyses of the DASS-anxiety subscale revealed poor postintervention reliability. However, given that one of the items measured mouth dryness, a common treatment side effect experienced by 40% of patients with advanced cancer [50], we removed this item and the reliability of the scale subsequently improved to an acceptable level (baseline Cronbach *α*=.80; after the intervention Cronbach *α*=.73).

*Spiritual well-being* was assessed using the Functional Assessment of Chronic Illness Therapy-Spiritual Well-being (FACIT-Sp-12) scale [51]. The scale assesses 3 domains of spiritual well-being (faith, meaning, and peace) and has 12 items rated on a 5-point Likert scale, ranging from 0 (“not at all”) to 4 (“very much”). A total of 2 items were reverse scored, and the sum of the items provided a total score that ranged from 0 to 48. Higher scores indicate greater spiritual well-being. Good internal reliability (Cronbach *α*=.81-.91) [51] and good factorial validity (*r*=0.7) have been shown among patients with cancer [52]. The measure demonstrated good internal reliability in this study at baseline (Cronbach *α*=.90) and after the intervention (Cronbach *α*=.94).

### Statistical Analyses

Data were analyzed using SPSS Statistics (version 26; IBM Corp). Descriptive statistics were conducted using numbers and percentages for categorical variables and means and SDs or medians and IQRs for continuous measures. The normality of all continuous outcome measures was assessed visually using the Shapiro-Wilk test. Square root transformation improved the normality of the DASS-depression and DASS-anxiety scales, and the EQ-5D-5L index improved when squared. The transformed data for these measures were used for all analyses. Linear mixed-effects models assessed whether baseline connection to nature (INS) was associated with quality of life (EQ-5D-5L), physical symptoms (FACIT-fatigue and BPI-SF), and psychological well-being (DASS-depression, DASS-anxiety and FACIT-Sp-12) and whether improvements over time in these metrics differed between those who had a weaker connection and those who had a stronger connection with nature at baseline. Time, baseline connection to nature, and the interaction between time and connection with nature were entered as fixed factors, with a random effect to account for repeated measures within the participants. Post hoc tests were used to compare the average change over time within the weaker and stronger baseline connection with nature groups, where there was an indication of a potential interaction (using a threshold of interaction *P* value of <.10). *P* values of <.05 were considered statistically significant. Adjustments for multiple testing were not performed because of the exploratory nature of this study.

## Results

### Overview

The participants in this study were all female (38/38, 100%), mostly New Zealand European (31/38, 82%), and had a median age of 51 years (Table 1). The majority did not work in paid employment (21/38, 55%) and were either married or living with a partner (22/38, 58%). The median time since cancer diagnosis was 5 years (compared with 2 years in the broader population with MBC [8]), and participants were currently undergoing a variety of cancer treatments at the time of study involvement. Most participants had a weaker compared with a stronger connection with nature at the baseline (29/38, 76%).

**Table 1:** Baseline characteristics of the sample (N=38).

Measure			Participants
Age (years), median (IQR)			51 (58-45)
**Ethnicity, n (%)**			
	New Zealand European	31 (82)	
	New Zealand Maori	6 (16)	
	Pacific	1 (3)	
**Highest education, n (%)**			
	Secondary	16 (42)	
	Tertiary	15 (40)	
	Postgraduate	7 (18)	
**Employment status, n (%)**			
	Full-time	10 (26)	
	Part-time	7 (18)	
	Not working	21 (55)	
**Relationship status, n (%)**			
	Single	7 (18)	
	Divorced or separated or widowed	9 (24)	
	Married or cohabitating	22 (58)	
**Current cancer treatment, n (%)**			
	Chemotherapy only	8 (21)	
	Hormone therapy only	16 (42)	
	Hormone and target therapy	8 (21)	
	Radiation and hormone therapy	1 (3)	
	No current cancer treatment	5 (13)	
Time since diagnosis (years), median (IQR)			5 (7)
**Connection with nature, mean (SD)**			3.95 (1.97)
	Weaker (scores 1-5), n (%)	29 (76)	
	Stronger (scores 6-7), n (%)	9 (24)	

### Assessment of Baseline Connection With Nature

#### Overview

**Figure 2:**
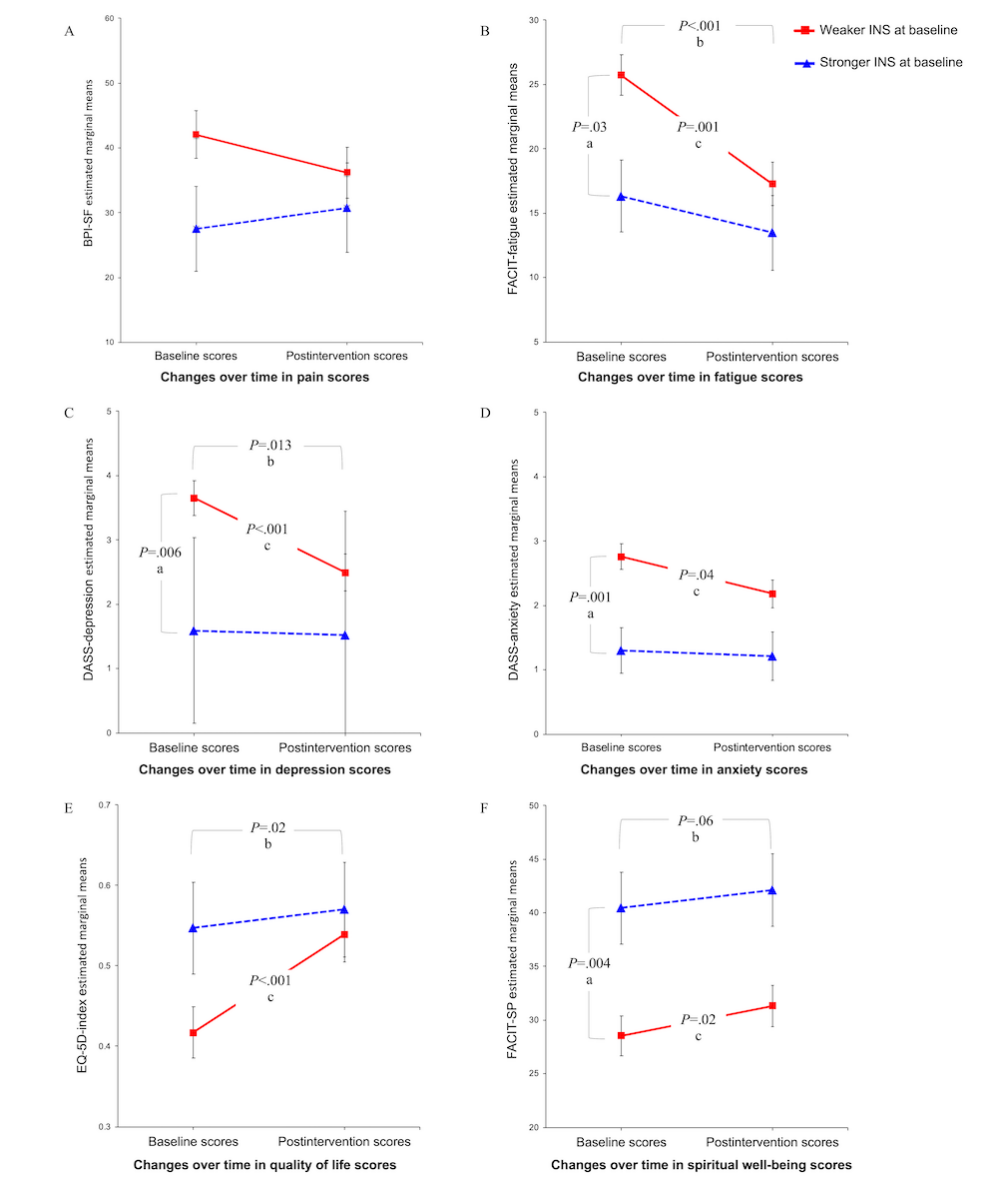
Mean scores of outcome measures over time by baseline connection with nature with SE bars. (A) Significant difference between weaker and stronger INS at baseline indicated in B, D, and F; (B) significant improvement over time indicated in B, C, E and F; (C) significant improvement between baseline and postintervention scores in participants with weaker INS at baseline indicated in B, C, D, E, and F. BPI-SF: Brief Pain Inventory Short Form; DASS: Depression, Anxiety, and Stress Scale; FACIT: Functional Assessment of Chronic Illness Therapy; FACIT-Sp-12: Functional Assessment of Chronic Illness Therapy-Spiritual Well-being scale; INS: Inclusion of Nature in the Self.

The linear mixed-effects models investigating the role of connection with nature indicated that the group with a weaker (cf stronger) connection with nature at baseline had poorer functioning on several metrics (fatigue, depression, anxiety, and spirituality; Table 2 and Figure 2). There was only one significant interaction between connection with nature and time, indicating that initial differences in depression among groups became less marked after the intervention, although a similar trend was observed in fatigue and quality of life scores. The results are discussed in more detail in further sections.

**Table 2:** Comparison of outcome measurements for time, baseline Inclusion of Nature in the Self (INS), and the interaction between time and baseline INS.

Measure and comparison			Estimated marginal mean difference (95% CI)		*P* value
**Brief Pain Inventory Short Form-pain**					
	Post- vs preintervention scores	1.31 (−6.55 to 9.17)		.74	
	Weak vs strong baseline INS	9.97 (−3.61 to 23.54)		.15	
	Time x weak baseline INS	−5.88 (−13.69 to 1.93)		.14	
	Time x strong baseline INS	3.27 (−10.38 to 16.91)		.63	
**Functional Assessment of Chronic Illness Therapy-fatigue**					
	Post- vs preintervention scores	5.66 (2.59 to 8.73)		.001	
	Weak vs strong baseline INS	6.60 (0.66 to 12.53)		.03	
	Time x weak baseline INS	−8.46 (−11.51 to −5.41)		<.001	
	Time x strong baseline INS	−2.86 (−8.18 to 2.46)		.28	
**Depression, Anxiety, and Stress** **Scale-depression**					
	Post- vs preintervention scores	0.62 (0.14 to 1.09)		.01	
	Weak vs strong baseline INS	1.52 (0.47 to 2.57)		.01	
	Time x weak baseline INS	−1.16 (−1.64 to −0.68)		<.001	
	Time x strong baseline INS	−0.07 (−0.90 to 0.76)		.86	
**Depression, Anxiety, and Stress** **Scale-anxiety**					
	Post- vs preintervention scores	0.33 (−0.21 to 0.87)		.23	
	Weak vs strong baseline INS	1.21 (0.55 to 1.87)		.001	
	Time x weak baseline INS	−0.57 (−1.11 to −0.04)		.04	
	Time x strong baseline INS	−0.09 (−1.03 to 0.86)		.85	
**EQ-5D-5L**					
	Post- vs preintervention scores	−0.07 (−0.13 to −0.01)		.02	
	Weak vs strong baseline INS	−0.08 (−0.20 to 0.04)		.19	
	Time x weak baseline INS	0.12 (0.06 to 0.18)		<.001	
	Time x strong baseline INS	0.02 (−0.08 to 0.13)		.66	
**Functional Assessment of Chronic Illness Therapy-Spiritual Well-being**					
	Post- vs preintervention scores	−2.24 (−4.58 to 0.11)		.06	
	Weak vs strong baseline INS	−11.37 (−18.81 to −3.92)		.004	
	Time x weak baseline INS	2.76 (0.42 to 5.10)		.02	
	Time x strong baseline INS	1.71 (−2.36 to 5.77)		.40	

#### Pain

Analyses of the effects of study involvement on pain indicated no main effects of time or baseline INS on the BPI-SF scores. Pain did not change over time (*F*_1,31.43_=0.12; *P*=.74) and did not vary according to baseline connection with nature (baseline INS: *F*_1,34.88_=2.22; *P*=.15), and there was no interaction effect between time and baseline INS (*F*_1,31.43_=1.41; *P*=.25; Figure 2A).

#### Fatigue

There was an effect of baseline connection with nature (*F*_1,35.38_=5.08; *P*=.03), in that women with weaker baseline INS scores had higher fatigue (mean 21.50, SE 1.43) than those with stronger INS scores (mean 14.90, SE 2.55). There was a main effect of time on fatigue (FACIT-Fatigue scores: *F*_1,31.45_=14.16; *P*=.001) where postintervention fatigue (mean 15.37, SE 1.68) was significantly lower than baseline fatigue (mean 21.03, SE 1.61; Figure 2B). However, the interaction between time and connection with nature indicated a trend (*F*_1,31.45_=3.45; *P*=.07), with post hoc tests revealing that fatigue levels only improved in women with weaker INS scores at baseline (FACIT-fatigue scores: baseline mean 25.72, SE 1.57; postintervention scores: mean 17.27, SE 1.67; *P<*.001). The FACIT-fatigue scores did not change in participants with a stronger baseline connection with nature (baseline mean 16.33, SE 2.81; postintervention mean 13.47, SE 2.92; *P*=.28).

#### Depression

There was a significant interaction between time and connection with nature on depression scores (DASS-depression: *F*_1,31.44_=5.35; *P*=.03 with cancer; Figure 2C). Only those with a weaker connection with nature at baseline had significant improvements in depression over time (weaker INS: baseline mean 3.65, SE 0.27; postintervention mean 2.49, SE 0.29; *P*<.001). In contrast, those with a stronger baseline connection with nature showed no difference over time (stronger INS: baseline mean 1.59, SE 0.48; postintervention mean 1.52, SE 0.50; *P*=.86). There was also an effect of baseline connection with nature (*F*_1,35.82_=8.55; *P*=.006) in that women with a weaker baseline connection with nature had greater depression (mean 3.07, SE 0.25) than those with a stronger connection to nature (mean 1.55, SE 0.45).

#### Anxiety

There was no overall effect of time on anxiety (DASS-anxiety: *F*_1,33.01_=1.53; *P*=.23); however, there was an effect of baseline connection with nature (*F*_1,34.39_=13.93; *P*=.001), where women with a weaker baseline connection to nature had significantly higher anxiety (mean 2.47, SE 0.16) than those with a stronger connection with nature (mean 1.26, SE 0.28; Figure 2D). The interaction between time and connection with nature was not significant (*F*_1,33.01_=0.82; *P=*.37).

#### Quality of Life

There was a main effect of time on quality of life (EQ-5D-5L index scores: *F*_1,31.07_=6.12; *P*=.02) such that quality of life after the intervention (mean 0.55, SE 0.03) was significantly greater than that at baseline (mean 0.48, SE 0.03). There was no effect of the baseline connection with nature on quality of life (*F*_1,35.19_=1.83; *P*=.19; Figure 2E), and the interaction between time and connection with nature indicated a nonsignificant trend (*F*_1,31.07_=2.87; *P*=.10). However, post hoc tests revealed that only participants with a weaker INS at baseline experienced improvements in quality of life over time (weaker INS: baseline mean 0.42, SE 0.03; postintervention mean 0.54, SE 0.03; *P*<.001 and stronger INS: baseline mean 0.55, SE 0.06; postintervention mean 0.57, SE 0.06; *P*=.66).

#### Spirituality

The effect of time on spirituality also indicated a trend but was not significant (FACIT-Sp-12: *F*_1,30.78_=3.78; *P*=.06). However, there was an effect of baseline connection with nature (*F*_1,35.99_=9.58; *P*=.004) where women with a stronger baseline connection with nature (mean 41.30, SE 3.21) had significantly greater spirituality than those with a weaker connection with nature (mean 29.93, SE 1.79; Figure 2F). There was no interaction between time and connection with nature (*F*_1,30.78_=0.21; *P*=.65).

#### Sensitivity Analyses

We evaluated the impact of splitting the INS at other points based on the aforementioned results. First, we dichotomized the scores as weak (1-4; 23/38, 61%) and strong (5-7; 15/38, 39%) and then trichotomized the scores as weak (1-3; 16/38, 42%), medium (4-5; 13/38, 34%), and strong (6-7; 9/38, 24%). The results of these models were essentially unchanged, except for the *P* values for the interaction of time and INS on depression, quality of life, and fatigue, which increased above the threshold of *P*<.10. Although post hoc tests in these instances remained consistent with the aforementioned results and continued to be strongly statistically significant (ie, *P*<.001), the statistical justification to report on these tests was diminished without the interaction effect.

## Discussion

### Principal Findings

This study investigated whether VR nature interventions might benefit women with MBC who are disconnected with nature. Primary analyses of this intervention found no differences in outcomes between the 2 VR nature experiences [33]; hence, this study focused on whether these interventions might provide differential benefit to women who were not strongly connected with nature at baseline. In line with the primary report [39], time effects revealed that participants reported significantly less fatigue, less depression, and a greater quality of life following the interventions compared with baseline. The difference in spirituality across time indicated a trend for improvement, although it did not meet the threshold for significance. Of note, our analyses revealed 2 key findings specific to our research focus on the connection with nature. First, our results demonstrated differences in well-being between those who had a weaker connection and those who had a stronger connection with nature, that is, women with a weaker connection with nature reported greater fatigue, depression, anxiety, and poorer spirituality than their strongly connected counterparts. Second, we also found evidence of a potential moderating effect between connection with nature and time on depression; only those with a weaker baseline connection with nature at baseline had improvements in depression following the intervention. Although similar patterns were observed for fatigue and quality of life, these effects did not reach the threshold for significance. In the following sections, we discuss the implications of these findings and consider how this report may inform future research in this area.

### Interpretation and Clinical Implications

This report extends primary analyses demonstrating the benefits of VR nature experiences [39] by indicating that patients with MBC, who are disconnected with nature have poorer well-being according to physical (fatigue) and psychological (depression, anxiety, and spirituality) metrics compared with women who are well connected with nature. In addition, although the effect was small (*P*=.03), our VR nature intervention was associated with improvements in depression among women who were disconnected with nature. The trend that these VR nature experiences might also be helpful for quality of life and fatigue in this population requires further investigation. Thus, the first contribution to the literature of this report lies in demonstrating a positive cross-sectional relationship between connection with nature and well-being in patients with MBC. Compared with those already strongly connected with nature, patients with MBC, with a relatively weaker connection reported poorer physical status (greater fatigue, although no differences in pain) and psychological function (greater depression and anxiety and poorer spiritual well-being). These results indicate that feeling connected with nature seems to matter in this population much like it does in other groups [53].

It is worth emphasizing that we focused on “connection” with nature (ie, asking participants how “close” they felt with nature) rather than “time” spent in nature. Recent work has demonstrated that it is not time spent in nature per se that is the critical factor for well-being. Instead, it is feeling connected or engaged with nature that is a key predictor in explaining variance in mental health and well-being [54]. Thus, activities that encourage engagement or connection are likely beneficial. It is also important to note that our study design limits the conclusions on the direction of the nature–well-being relationship. It is possible that rather than disconnection with nature leading to poorer physical and psychological status in patients with MBC, the reverse might be true, such that poorer mental or physical health inhibits connection with nature. We suspect that the relationship is bidirectional, much like the exercise–well-being relationship [55]; that is, connection with nature positively affects well-being, and positive well-being makes a person more likely to connect with nature. These findings have important implications for supporting women with MBC. Further investigation into the direction and nature of this relationship is warranted, including the extent to which feelings of *dis*connectedness are stable over time (ie, trait dispositions) versus fluctuate in response to short-term (ie, state) situations.

The second contribution of this work lies in demonstrating that a nature-based intervention might provide particular benefits to women who are disconnected with nature. Consistent with well-established evidence that describes how exposure to natural environments benefits groups who typically have infrequent contact with nature (eg, urban dwellers) [56-58], this report reveals that the participants in our study most likely to benefit were those who initially felt disconnected with nature. A burgeoning body of work has established that green spaces and activities such as forest bathing can provide both psychological benefits (stress reduction and mood improvement [56]) and physiological benefits, including reductions in blood pressure and heart rate [59,60] and improvements in immune function [61,62]. Benefits such as these are relevant to populations with cancer, where disease trajectories and quality of life might be improved through enhanced physiological and psychological functions. Although the benefits revealed in this study were limited to improvements in depression, similar patterns were observed in fatigue and quality of life, and these areas appear worthy of future attention. This work extends previous literature that has primarily focused on urban dwellers by indicating that a clinical population who is disconnected with nature owing to constraints that are either medical (eg, cancer) and psychological (eg, depression) might also benefit.

Finally, our findings suggest that *virtual* exposure to nature may be sufficient to generate benefits. Virtual experiences may be important in contexts in which patients with cancer are tied to urban settings that provide their treatment or indoors because of physical or psychological constraints. Virtual exposure to nature might provide benefits that align with work in other clinical contexts demonstrating benefits for pain management, stroke rehabilitation, and distraction during cancer treatment [25]. As noted earlier, research has demonstrated that a virtual replication of a nature experience provides almost identical benefits (physiological arousal, mood, and restorativeness) to the real-world experience [26]. Furthermore, following from the earlier point that connection rather than time in nature matters most, VR interventions appear particularly well placed to offer interactive activities designed to foster connectedness and active engagement with nature. Therefore, rather than simply providing an opportunity to observe (ie, be a bystander), virtual nature-based activities that encourage engagement may be helpful. Furthermore, interactive experiences may not need to be lengthy in a “quality over quantity”–type approach, and investigation of this possibility is warranted.

Our findings have important clinical implications for patients with MBC, a population that is often overlooked. Numerous studies have reported that the psychological and physical needs of patients with advanced cancer are frequently unmet [63,64]. Simple, scalable interventions such as VR nature experiences seem worthy of future attention. VR interventions designed to stimulate feelings of connectedness with nature appear to have merits, and brief interventions may be sufficient. In the context of scarce resources and fierce competition for the health care dollar, these preliminary findings provide a general indication of where resources could be effectively targeted. VR interventions are relatively affordable and can be implemented in a person’s own home as well as in hospital or hospice care, making this an approach worthy of further consideration.

### Limitations and Suggestions for Future Research

Although this report is the first to provide evidence that a VR nature experience might be of particular benefit to women with MBC, who are disconnected from nature, this work is not without its limitations. First, it is worth emphasizing that this was a preliminary study with only a small number of participants (N=38), and as such, the study was only powered to identify large effects. The group with a higher connection to nature at baseline was small (9/38, 24%), and despite sensitivity analyses to determine the best way to categorize data, the statistical power to detect interaction effects was limited. Therefore, the results of this study should be interpreted as preliminary findings that require further investigation in other studies. Although our confidence in the merits of this intervention is bolstered by the fact that participants were compliant, enjoyed their experiences, and were generally open to the idea of using VR again [39], future work should recruit larger samples that will provide insight into the physical and psychological aspects of well-being most likely to be improved through an intervention of this kind.

In addition, as noted, this report outlines secondary analyses that did not assess the differences between the 2 VR experiences. Although it seems likely that different kinds of VR nature experiences might offer different types of benefits, primary analyses found no differences between the 2 interventions presented in this study [39]. Notably, there were numerous stylistic and content differences between the 2 VR experiences, and our design precludes comments on which of these elements might have been the most therapeutically potent. For instance, one experience used the real-life footage of nature scenes (Ripple), whereas the other was an animated experience (Happy Place). The latter included a greater number of interactive activities and thus, probably offered greater opportunities for distraction, but the former might have been more meditative. Furthermore, an alternative explanation for our findings is that those who perceived themselves as more connected to nature (ie, with higher INS scores) rejected the VR representations as oversimplifications of real nature compared with those with lower INS scores who were more satisfied with the simplistic representations of nature. Understanding how various characteristics of a VR nature intervention might influence outcomes and how people with varying degrees of self-perceived connection of nature seem worthy of investigation. Future studies could standardize aspects of the experience across conditions to assess, for instance, how the *sounds* of nature compare with the *sights* of nature, how *animated* footage compares with *real-world* photography, or how *guided relaxation* compares with *self-directed* experiences (to name a few). These are opportunities for future research to inform the development of targeted interventions.

Finally, this work is limited in that we did not include a control group, nor did we compare nature experience to a different kind of experience (eg, a gaming experience); thus, we cannot claim that the intervention or exposure to nature specifically caused benefits. However, some confidence that this might be the case is drawn from other evidence that VR nature experiences trump other virtual experiences [24] and our own participant feedback describing the therapeutic benefits of the experience, *“*Since starting the experiment I have had more energy, lasted full days at work, could still function when I got home, my memory is better ... it’s the best I’ve felt since before starting treatment*”* [39]*.* Confirming the causality of benefits with regard to VR interventions or the potency of nature-based activities requires further study. It might also be that an interactive VR nature experience that can be shared with children or grandchildren might offer incremental benefits given the well-established benefits of social interaction [65]. Accordingly, future studies should assess social support as a possible confounding or moderating factor. Finally, our findings may demonstrate the power of interventions to improve outcomes by providing support, attention, and care to vulnerable groups. Women with MBC certainly need psychosocial support, and it is possible that any intervention that provides focused attention would have led to benefits.

### Conclusions

This report is the first to provide preliminary evidence that feeling connected with nature is associated with better physical and psychological status in patients with MBC and that VR nature interventions might be of particular benefit for this clinical population. These findings have implications for the development of future interventions so that groups can be targeted not only where the need is most significant but also where benefits are most likely to be gained. For example, nature connectedness interventions could be developed for people who avoid venturing outdoors owing to clinically significant anxiety or depression. Such studies should focus on activities that encourage connection (rather simply exposure) with nature and investigate the aspects of VR nature interventions that have the greatest therapeutic potential.

## Abbreviations

BPI-SF: Brief Pain Inventory Short Form
DASS: Depression, Anxiety, and Stress Scale
DASS-21: Depression, Anxiety, and Stress Scale Short Form
FACIT: Functional Assessment of Chronic Illness Therapy
FACIT-Sp-12: Functional Assessment of Chronic Illness Therapy-Spiritual Well-being
INS: Inclusion of Nature in the Self
MBC: metastatic breast cancer
REDCap: Research Electronic Data Capture
VR: virtual reality
